# Nutritional status of pediatric patients with inflammatory bowel diseases is related to disease duration and clinical picture at diagnosis

**DOI:** 10.1038/s41598-023-48504-8

**Published:** 2023-12-02

**Authors:** Katarzyna Pawłowska-Seredyńska, Katarzyna Akutko, Wioleta Umławska, Bartłomiej Śmieszniak, Rafał Seredyński, Andrzej Stawarski, Tomasz Pytrus, Barbara Iwańczak

**Affiliations:** 1 Department of Human Biology, University of Wroclaw, Wroclaw, Poland; 2 2nd Clinical Department of Pediatrics, Gastroenterology and Nutrition, Medical University of Wroclaw, Wroclaw, Poland; 3 Department of Physiology, Medical University of Wroclaw, Wroclaw, Poland

**Keywords:** Gastrointestinal diseases, Nutrition, Paediatrics

## Abstract

This cross-sectional study presents the nutritional status of newly diagnosed pediatric patients with Crohn’s disease (CD) and ulcerative colitis (UC) and its association with the duration of the disease and selected clinical features. We analyzed the data of 41 pediatric patients with CD and 29 with UC (mean age: 13.1 y, range: 5.2–18.0 y) up to 3 mo. from diagnosis. Anthropometry included body weight, body height, body mass index (BMI), three skinfold thicknesses, mid-upper arm circumference and mid-upper arm muscle circumference adjusted for age and sex using national standards. Anthropometry was linked to the disease duration, location of the disease, symptoms, and blood test results. Both studied groups presented significantly lower BMI compared to the reference population, but only children with CD characterized with significantly worse nutritional status according to arm anthropometry. In CD, better nutritional status was associated mainly with longer disease duration and, to a lesser extent, with extraintestinal manifestations, perianal disease, and small intestinal lesions. In UC, anemia at diagnosis was associated with poor nutritional status. Our finding emphasizes the need for more attentive diagnostic care for pediatric patients who exhibit extraintestinal symptoms or perianal disease with no obvious signs of malnutrition, to avoid diagnostic delays.

Inflammatory bowel diseases (IBD), including Crohn’s disease (CD) and ulcerative colitis (UC), are chronic autoimmune disorders of the gastrointestinal tract. Whereas they may occur at any age, pediatric patients comprise about 20% of new IBD cases^1^. Epidemiological studies showed rapid increase in the incidence and prevalence of pediatric-onset IBD worldwide over the past 20 years, which is a challenge for a medicine of developmental period^2,3^.

Body weight loss or stagnation are common symptoms preceding the diagnosis of IBD, which has been reported in about 60–80% of newly diagnosed pediatric patients with CD and in about 40% of those with UC^4,5^. Poor nutritional status in IBD has been attributed to malabsorption, intestinal loss of energy and nutrients, dietary restrictions, lack of appetite, and to the altered nutrient metabolism and utilization^6^. Up to 35% of pediatric CD and up to 25% of pediatric UC are underweight at the time of diagnosis^4,6–10^. On the other hand, overweight and obesity have been increasingly more common in pediatric IBD, especially in children with UC, which is consistent with the trend observed worldwide^4,7,8,10–12^.

Body mass index (BMI) has been commonly used as a screening tool to assess the risk of malnutrition among children and adults. However, BMI often hides the problem of cachexia, especially in individuals with chronic illness^13,14^. Several DXA-based studies have revealed that lean body mass deficiency may affect even 30% of children and adolescents with CD and 10–15% of those with UC, regardless of the time from diagnosis^14–16^. Lean body and muscle mass deficiencies have been associated with malnutrition, reduced muscle synthesis, and increased protein degradation via the proinflammatory cytokines pathways^17^.

Lower occurrence of nutritional and growth impairments in pediatric UC compared to CD has been associated with less extensive lesions and the absence of the upper gastrointestinal tract involvement, along with more specific symptomology that facilitates early detection of the disease.^6,7^ Despite that, there is conflicting evidence in this regard, likely due to differences in selection criteria pertaining to comorbidities, treatment, or time elapsed since diagnosis. Hence, our study aims to evaluate the nutritional status of newly diagnosed pediatric patients with CD and UC through the anthropometric assessments and its association with the duration of the disease and selected clinical features of the diseases.

## Material and methods

### Study protocol

We collected detailed anthropometric data of children with IBD diagnosed and treated in the 2nd Clinical Department of Pediatrics, Gastroenterology and Nutrition (Wroclaw, Poland) between October 2012 and March 2020. The study protocol conformed to the ethical guidelines of the Helsinki Declaration and was approved by the local Ethical Committee of Wroclaw Medical University, Poland (KB 199/2013). All caregivers of studied patients and patients ≥ 16 y of age gave their written informed consent to anthropometric measurements and to use their clinical data in the study.

### Study group selection

**Figure 1:**
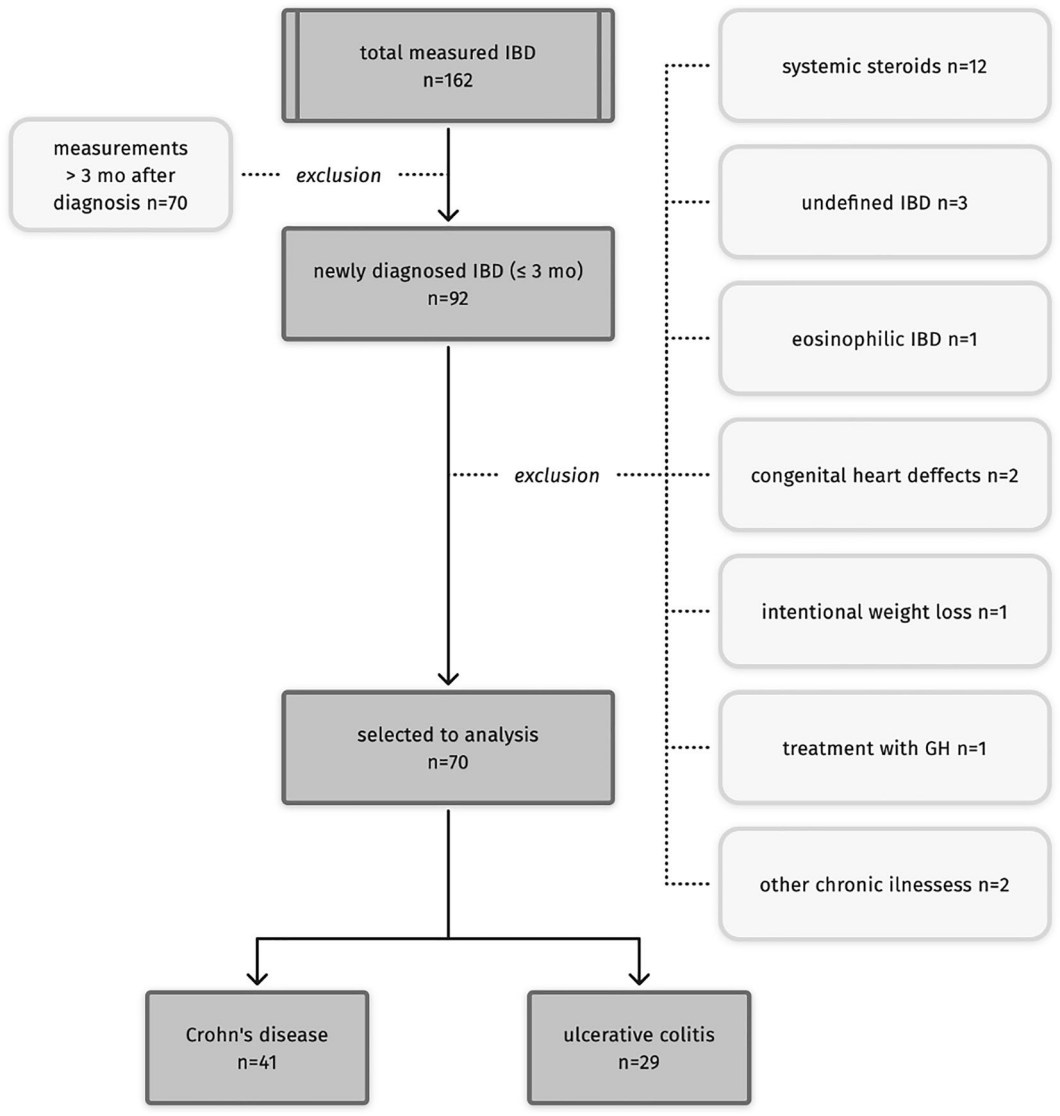
Selection of patients.

91 patients (out of 162) with IBD were measured within 3 months from endoscopic examination confirming IBD. We included the data of patients who: were not diagnosed with any condition, or used any medication (i.e., systemic corticosteroids), affecting physical growth or body composition, did not declare any weight loss diet. Finally, the data of 70 patients (mean age: 13.1 y, range: 5.2–18.0 y) with IBD were included in the analysis. Figure 1 presents the selection the data of patients to the analysis. None of the patients received any nutritional treatment nor dietitian support before the examination.

### Anthropometric assessment

All anthropometric measurements were performed at morning by a single trained investigator (KP-S). Patients were after overnight fast and lightly dressed. Body height was measured up to 0.1 cm with Martin’s anthropometer (Alumet Inc.). Body weight was measured up to 0.1 kg with electronic scale being a part of InBody J10 analyzer (Biospace Inc.). Three skinfold thicknesses (SFTs)—tricipital SFT, subscapular SFT and abdominal SFT—were measured up to 0.2 mm with Holtain caliper (Alumet Inc.). Mid-upper arm circumference (MUAC) was measured in the midway between acromion and ulnar processes up to 0.1 cm, using anthropometric tape (Seca Inc.). All measurements were performed in accordance with the methodology used in anthropology and described in Warsaw standards.

BMI was calculated as body weight (kg) divided by body height (m) squared. We also calculated the sum of three skinfold thicknesses (sum of 3 SFTs) and mid upper-arm muscle circumference (MUAMC) using calculation: *MUAC (cm)-(Pi x tricipital SFT (cm))*.

All measurements and anthropometric indices were standardized using Warsaw growth standards^18^ and presented as sex- and age- adjusted z-scores. The growth standards are based on the reference population of Warsaw children attending to randomly selected schools and preschools in years 1996–1999. Only healthy children, without congenital defects or developmental disorders, participated in the study.

Body height z-score <  − 1.64 was defined as growth failure, and body height z-score <  − 2 was defined as short stature. Underweight was defined as BMI z-score <  − 2, overweight—BMI z-score > 1 and ≤ 2, obesity—BMI z-score > 2. Lean body deficiency was defined as MUAMC z-score <  − 2.

### Clinical data

IBD was diagnosed according to current at the time guidelines (Porto or revised Porto criteria)^19,20^. The data regarding approximate date of first symptoms (used to calculate disease duration from the first symptoms to anthropometric examination in months, and the age of first symptoms in years), history of symptoms, location of lesions in gastrointestinal tract, the occurrence of perianal lesions, clinical activity of the disease, medication used, and other than IBD medical conditions were obtained from medical records. Extraintestinal manifestations (EIMs) of IBD included i.e., joint pain, skin lesions (as erythema nodosum), sclerosing cholangitis, eye lesions, unexplained fevers, or lethargy, and excluded growth and puberty disorders, and anemia to avoid autocorrelation in the analysis. The presence of mentioned extraintestinal symptoms and/or perianal (PA) disease (i.e., fissures, abscesses, fistulas) have been considered as one category of symptoms – ‘EIMs and PA disease’.

The involvement of the small intestine was established based on magnetic resonance enterography. Clinical activity of the disease was based on Pediatric Crohn’s Disease Activity Index (PCDAI) modified by Ryżko and Woynarowski in children with CD (Appendix 1, Supplementary Information)^21,22^ and Pediatric Ulcerative Colitis Index (PUCAI) in children with UC^23^. PCDAI score > 30 and PUCAI score ≥ 35 were considered moderate to severe disease activity^24,25^.

Blood test results we considered included high-sensitivity C-reactive protein (hsCRP, mg/dL), total protein (mg/dL), serum albumin (mg/dL), total cholesterol (mg/dL) and hemoglobin (mg/dL). Anemia was defined as low hemoglobin or hematocrit levels for sex and age according to the WHO criteria^26^ or the intake of iron supplement on doctor’s order. All blood analyses were performed in the Central Laboratory of the University Clinical Hospital in Wroclaw according to standard methods.

### Statistical analysis

Analyses were performed using STATISTICA 13 software (StatSoft Ltd., Krakow, Poland). Quantitative data normality was tested using Kolmogorov-Smirnoff test. Differences in quantitative data between two groups were tested using unpaired t-test or Mann–Whitney test. Differences between mean z-scores of anthropometric indices and reference population were tested with t-test for one sample. Differences in qualitative data distribution between groups were tested using χ^2^ test with Yates correction. The relationship between two quantitative variables was evaluated using Pearson’s correlation. Missing data were removed from some analyses using pairwise deletion. P-values below 0.05 were considered statistically significant.

For anthropometric indices of nutritional status (z-score) related significantly to more than one disease-related factor we performed two-factorial linear regression analyzes. Models included two types of predictors—quantitative and qualitative. Qualitative variables were dichotomous and coded as 0 (which meant ‘no’ or ‘the absence’) or 1 (which meant ‘yes’ or ‘the presence’). Models included only predictors which were not correlated with each other.

## Results

### Clinical and anthropometric characteristics of patients according to IBD type

Table 1 presents clinical characteristics of patients according to the diagnosis. Disease duration was significantly longer in children with CD than in children with UC. Moreover, EIMs and PA disease, and body weight loss as well, were more often noted in children with CD than in children with UC. This group was also characterized with higher serum hsCRP and lower serum albumin level.

**Table 1:** Clinical characteristics of patients with Crohn’s disease and patients with ulcerative colitis.

	Crohn’s disease (n = 41)	Ulcerative colitis (n = 29)	p-value
Boys	19/41 (46.3%)	16/29 (55.2%)	Ns (χ^2^)
Age (y)	13.35 ± 2.91	12.74 ± 2.93	Ns (t)
Age of first symptoms (y)	10.48 ± 4.27	11.96 ± 3.40	Ns (t)
Disease duration (mo)^a^	12.76, 5.72: 46.82	6.84, 2.99: 12.66	< 0.001 (t) ^b^
Time from diagnosis to measurement (mo)	0.55 ± 0.94	0.55 ± 0.91	Ns (Z)
EIMs and PA disease^c^ including:	23/40 (57.5%)	3/29 (10.3%)	-
EIMs	15/40 (37.5%)	3/29 (10.3%)	0.029 (χ^2^)^d^
PA disease	16/40 (40.0%)	0/29 (0.0%)	-
Body weight loss	25/41 (61.0%)	9/29 (31.0%)	0.026 (χ^2^)
Growth failure	7/41 (17.1%)	2/29 (6.9%)	Ns (χ^2^)
Short stature	3/41 (7.3%)	1/29 (3.4%)	Ns (χ^2^)
Underweight Normal weight Overweight Obesity	5/41 (12.2%) 33/41 (80.5%) 3/41 (7.3%) 0/41 (0.0%)	2/29 (6.9%) 24/19 (82.8%) 2/29 (6.9%) 1/30 (3.5%)	Ns (χ^2^)
Lean body deficiency	6/41 (14.6%)	1/29 (3.4%)	-
Disease activity:			
Remission to mild Moderate to severe	24/39 (61.5%) 15/39 (38.5%)	15/23 (65.2%) 8/23 (34.8%)	-
PCDAI/PUCAI	25.8 ± 16.4	31.94 ± 23.13	-
Medication use (yes)	16/41 (39.0%)	12/29 (41.4%)	Ns (χ^2^)
hsCRP (mg/dL)	21.49 ± 26.59	7.45 ± 15.21	0.002 (t)^c^
Serum albumin (mg/dL)	3.87 ± 0.44	4.17 ± 0.44	0.014 (t) ^c^
Total protein (mg/dL)	7.04 ± 0.69	7.21 ± 0.63	Ns (t)
Total cholesterol (mg/dL)	150.77 ± 26.90	150.84 ± 34.12	Ns (t)
Hemoglobin (mg/dL)	11.67 ± 1.73	11.68 ± 1.70	Ns (t)
Anemia	25/30 (62.5%)	19/29 (65.5%)	Ns (χ^2^)

*CD* Crohn’s disease, *EIMs* extraintestinal manifestations, *hsCRP* high-sensitivity C-reactive protein, *mo* months, *PA* perianal, *PCDAI* Pediatric Crohn’s Disease Activity Index, *PUCAI* Pediatric Ulcerative Colitis Activity Index, *UC* ulcerative colitis, *y* years.

Quantitative data was presented as mean ± SD, except for disease duration. Qualitative data was presented as number and % of cases in the group.

^a^Median (Me) and interquartile range (IQR).

^b^Analysis based on logarithm data.

^c^Symptoms including extraintestinal manifestations (without anemia and disorders of growth, nutritional status, and puberty) and perianal disease.

^d^The analysis concerns only extraintestinal manifestations.

χ^2^—p-value according to χ^2^test with Yates’ correction.

t—p-value according to unpaired t-test.

Z—p-value according to U Mann–Whitney test.

**Figure 2:**
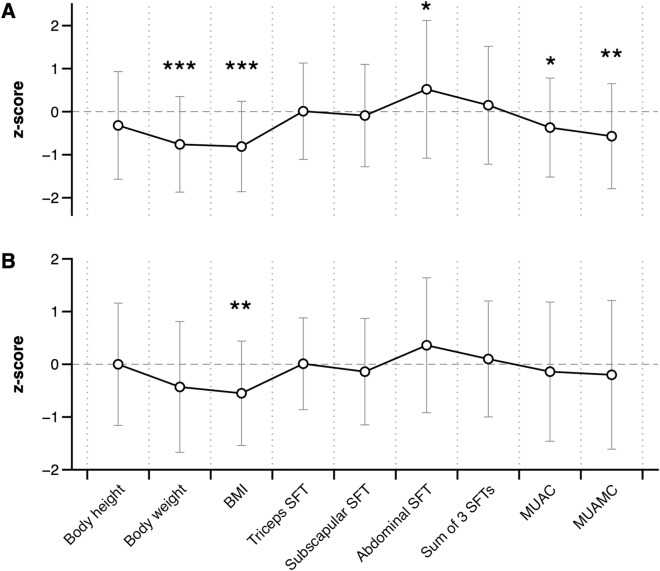
Mean values (+ / − SD) of sex- and age-adjusted anthropometric indices (z-scores) of children with Crohn’s disease (**A**) and ulcerative colitis (**B**) and statistical significances of differences in anthropometry between patients and reference population according to one-sample t-test (*p < 0.05, **p < 0.01, ***p < 0.001).

Figure 2a and b show deviations of anthropometric indices (z-scores) in CD and UC groups from sex- and age-matched reference population we used. In children with CD, we noted significantly lower body weight ( − 0.76 ± 1.11), BMI ( − 0.81 ± 1.05), MUAC ( − 0.37 ± 1.15) and MUAMC ( − 0.57 ± 1.22) and greater abdominal SFT (0.52 ± 1.60) compared to the standard. In children with UC only BMI ( − 0.55 ± 0.99) deviated significantly from the standard. However, we did not find any differences in z-scores of anthropometric indices between both studied groups.

### Nutritional status in Crohn’s disease

Disease duration positively correlated with z-scores of body weight (r = 0.38, *p* = 0.017), BMI (r = 0.42, *p* = 0.007), tricipital SFT (r = 0.43, *p* = 0.006), subscapular SFT (r = 0.57, *p* < 0.001), and the sum of SFTs (r = 0.42, *p* = 0.007) in patients with CD. We also noted that the age of patients with CD positively correlated with abdominal SFT (r = 0.40, *p* = 0.009) and the sum of SFTs (r = 0.42, *p* = 0.042), and the age of first symptoms negatively correlated with BMI z-score (r =  − 0.37, *p* = 0.022). All correlations between anthropometry and quantitative clinical data were presented in supplementary Table I (Supplementary Information).

**Figure 3:**
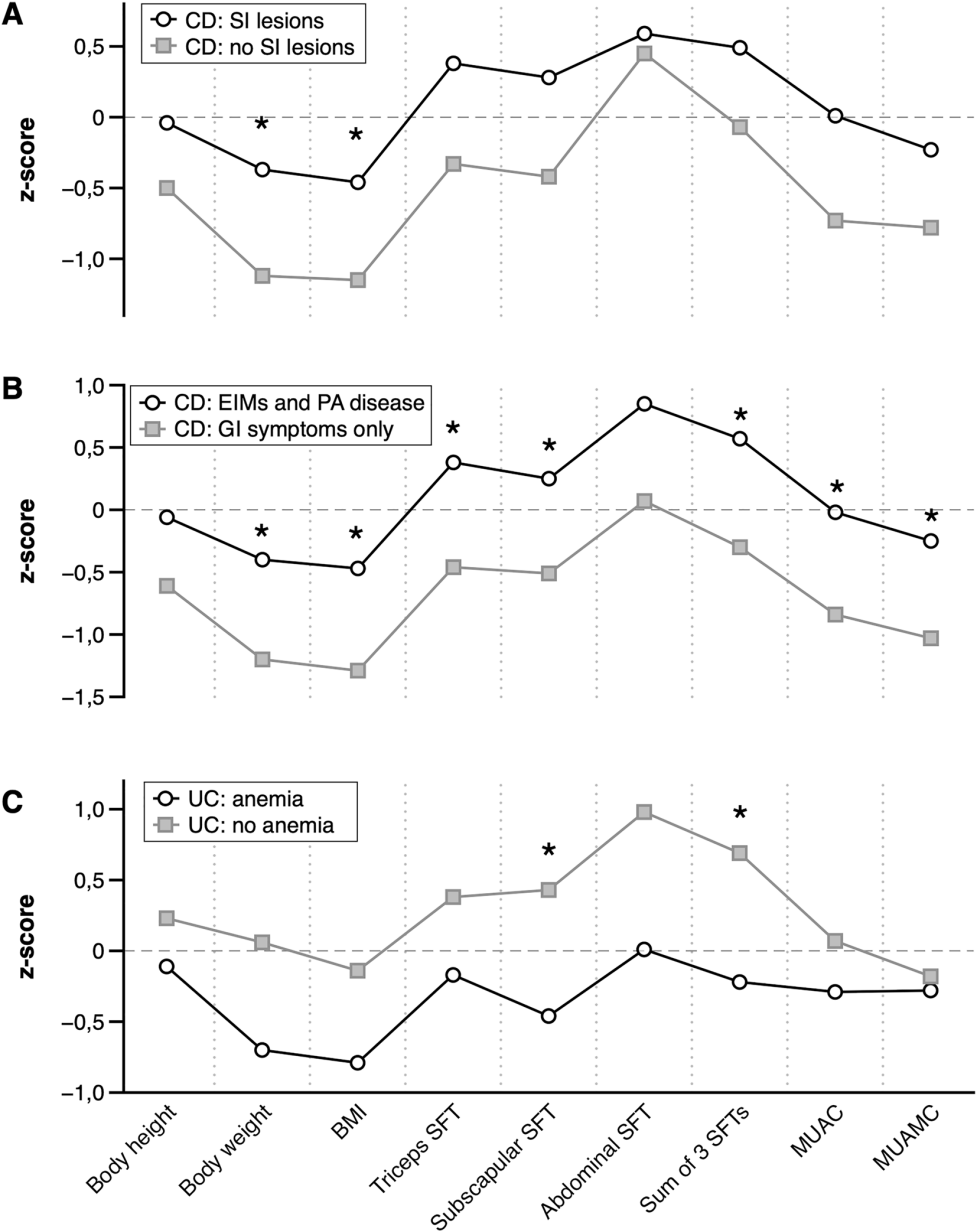
Mean values of sex- and age-adjusted anthropometric indices (z-scores) of: children with Crohn’s disease according to disease location (**A**), children with Crohn’s disease according to the type of symptoms (**B**), and children with ulcerative colitis regarding anemia (**C**) and statistical significances of differences in anthropometry between groups according to unpaired t-test or Mann-Whitney test (*p < 0.05, **p < 0.01, ***p < 0.001; *CD* Crohn’s disease, *EIMs and PA disease* extraintestinal manifestations and perianal disease, *GI* gastrointestinal, *SI* small intestine, *UC* ulcerative colitis).

In CD, anthropometry was also related to two qualitative factors—small intestinal location of lesions and the presence of EIMs and PA disease (Fig. 3a,b). Children with lesions in the small intestine presented significantly greater z-scores of body weight and BMI and tended to have greater tricipital SFT, and MUAC. Moreover, children with the disease in the small intestine noted body weight loss before diagnosis slightly less often than children without lesions in this location (52.4% vs 76.5%, *p* = 0.233). Children presenting EIMs and PA disease were also heavier (according to body weight and BMI z-scores), more adipose (according to tricipital, subscapular and sum of SFTs z-scores) and had less lean body deficits (according to MUAC and MUAMC z-scores) than children with only gastrointestinal symptoms. The frequencies of body weight loss before diagnosis were similar in both groups (69.6% in EIMs and PA disease group vs 52.9% in the second group, *p* = 0.457) The exact values (mean ± SD) for children with CD regarding disease location and kind of symptoms are presented in supplementary Tables IIa and b (Supplementary Information).

Children with lesions in the small intestine characterized with longer disease duration from the first symptoms (median 26.8 mo. vs 8.1 mo., *p* = 0.017). There was also a tendency to longer disease duration in CD children with EIMs and PA disease compared to those with only symptoms from the gastrointestinal tract (median 24.0 mo. vs 11.9 mo., *p* = 0.170). Moreover, EIMs and PA disease were twice more often observed in children with CD located in the small intestine than in children without lesions in the small intestine (76.2% vs 38.9% respectively, *p* = 0.028).

For BMI z-score at diagnosis we were able to build three models including two independent predictors, and for other anthropometric indicators we built only one model including disease duration (mo) and the presence of EIMs and PA disease as predictors. Performed analyzes showed that BMI, tricipital SFT, subscapular SFT and the sum of three SFTs z-scores were significantly related to disease duration when controlling the presence of EIMs and PA disease (model 1 for each explained variable). These associations were positive, which means that the longer disease duration, the better nutritional status right after diagnosis. Both predictors explained between 18.7 and 31.8% of the variance of described anthropometric parameters. Model 3, including the age of the first symptoms and the presence of EIMs and PA disease, indicated the significance of both tested predictors, and the model 3 explained 19.6% (*p* = 0.007) of BMI z-score variation in the group of newly diagnosed CD. In this model, each year of age was associated with a decrease in BMI z-score of 0.08 units, and the presence of EIMs and PA disease was related to the decrease in BMI z-score of 0.31 units. Results of all regression analyzes were presented in Table 2.

**Table 2:** Two-factorial linear regression analyses explaining the relationships between anthropometric indices of nutritional status (z-scores) right after Crohn’s disease diagnosis and: disease duration from the first symptoms (mo), age of the first symptoms (y), the presence of lesions in the small intestine, and the presence of extraintestinal manifestations and perianal disease.

Model	Predictors	B	95% CI	β	95% CI	t	p	Adjusted R^2^	p
Dependent variable: body weight (z-score)									
Model 1 N = 39	Disease duration (mo)	0.01	0.00 - 0.02	0.31	-0.01 - 0.62	1.99	0.054	0.168	0.014
	EIMs and PA disease^a^	-0.31	-0.66 - 0.04	-0.27	-0.58 - 0.04	-1.75	0.088		
Dependent variable: BMI (z-score)									
Model 1 N = 39	Disease duration (mo)	0.01	0.001 - 0.02	0.34	0.04 - 0.065	2.30	0.027	0.215	0.005
	EIMs and PA disease^a^	-0.31	-0.64 - 0.01	-0.29	-0.59 - 0.01	-1.94	0.060		
Model 2 N = 37	Age of the first symptoms (y)	-0.07	-0.15 - 0.01	-0.28	-0.60 - 0.05	-1.73	0.093	0.136	0.031
	Small intestinal lesions^a^	-0.29	-0.63 - 0.06	-0.27	-0.59 - 0.05	-1.70	0.098		
Model 3 N = 39	Age of the first symptoms (y)	-0.08	-0.15 - -0.001	-0.31	-0.61 - -0.01	-2.07	0.045	0.196	0.007
	EIMs and PA disease^a^	-0.36	-0.68 - -0.03	-0.33	-0.63 - -0.03	-2.23	0.032		
Dependent variable: tricipital SFT (z-score)									
Model 1 N = 39	Disease duration (mo)	0.01	0.001 - 0.02	0.36	0.06 - 0.66	2.41	0.021	0.213	0.005
	EIMs and PA disease^a^	-0.31	-0.66 - 0.04	-0.27	-0.57 - 0.03	-1.80	0.081		
Dependent variable: subscapular SFT (z-score)									
Model 1 N = 39	Disease duration (mo)	0.01	0.01 - 0.02	0.52	0.24 - 0.80	-2.93	0.006	0.318	< 0.001
	EIMs and PA disease^a^	0.17	-0.57 - 0.12	-0.18	-0.47 - 0.10	-1.31	0.198		
Dependent variable: sum of SFTs (z-score)									
Model 1 N = 39	Disease duration (mo)	0.01	0.002 - 0.02	0.36	0.05 - 0.67	2.37	0.023	0.187	0.009
	EIMs and PA disease^a^	-0.32	-0.75 - 0.12	-0.23	-0.54 - 0.08	-1.52	0.136		

*BMI* body mass index, *EIMs* extraintestinal manifestations, *mo* months; *PA* perianal, *SFT* skinfold thickness, *y* years.

^a^ ‘Yes’ coded as 0, ‘no’ coded as 1.

### Nutritional status in ulcerative colitis

In children with UC only the anemia was associated with lower subcutaneous fat content measured with subscapular SFT and sum of SFTs z-scores. Children with anemia presented also slightly lower BMI z-score than those without anemia (*p* = 0.062) (Fig. 3c and Table IIc (Supplementary Information)). Body weight loss was reported in 42.1% of children with anemia and in 10.0% of children without anemia (*p* = 0.176, low expected frequencies). Children with UC who noted body weight loss were older (14.9 y vs 11.8 y, *p* = 0.014) and had their first symptoms at the older age (14.2 y vs 10.9 y, *p* = 0.015) than children who did not lose their body weight.

There was no correlation between any of quantitative data and z-scores of anthropometric indices in children with UC. In this group, we observed only positive correlation of serum albumin level with total protein level (r = 0.58, *p* = 0.001) and slightly with hemoglobin level (r = 0.37, 0.050), and negative correlation with hs CRP level ( − 0.44, p = 0.016) and PUCAI score ( − 0.74, *p* < 0.001). All correlations were presented in supplementary Table III (Supplementary Information).

## Discussion

According to our study, children newly diagnosed with CD or UC present poor nutritional status regarding body weight and BMI compared to the reference population. CD patients, compared to UC ones, were characterized by more severe disease course, embodied in two times longer disease duration, three times more frequent EIMs, greater inflammatory status, and twice more common body weight loss before the diagnosis. Despite that, we found no significant differences in the sex- and age-adjusted anthropometric indices between both studied groups. Although nutritional status of children newly diagnosed with IBD was described in many previous reports^4,14,27^, most of them did not consider that disease-related factors affecting nutritional status may depend on disease entity. In our study, nutritional status of pediatric patients with newly diagnosed CD and UC was related to disease course in different ways.

We found the underweight twice more common in CD than in UC pediatric patients (12.6% and 6.7% respectively), which is consistent with other reports^4,6–10^. Lean body deficits (MUAMC <  − 2 SDS) were also more prevalent in CD than in UC. Although mean z-scores of MUAMC did not differ between patients CD and UC, only children with CD were characterized with significantly lower MUAMC compared to reference population of healthy children. Similarly, Sila and coworkers showed slightly greater deficits in anthropometry, body composition, and handgrip strength in CD patients compared to the UC ones. Nevertheless, significant differences in total energy, protein, and fat intakes were observed only between children with UC and healthy controls^8^. Poor nutritional status in CD has been commonly linked to the malabsorption of nutrients due to the small intestinal inflammation^15,28^, which might explain relatively frequent underweight despite normal intake of nutrients^8^. In our CD group, lesions in the small intestine were related to greater body weight and BMI z-scores at diagnosis, but this relationship might be due to some confounding factors. Two-factorial analysis showed that small intestinal location of CD slightly lowered BMI z-score when the age of first symptoms was controlled. Burnham and colleagues suggested a relationship between lean body deficiency and inflammation in the small intestine based on the administration of mesalamine,^15^ but recent studies did not confirm such observation^17,27,29^.

While IBD remains routinely associated with underweight and wasting condition, the prevalence of the excessive body weight among IBD children is rising^6^. It is likely due to more frequent overweight and obesity observed in pediatric population worldwide, as well as relatively earlier diagnosis. In fact, according to multiple reports, the prevalence of overweight and obesity in newly diagnosed IBD proportionally related to the prevalence observed in general population, being about two to three times higher among UC children compared to CD^4,7,8,10–12^. In our study population, prevalence of the excessive body weight in both groups was comparable (7.5% in CD and 10% in UC) and was about two times lower than the value reported for general population of Polish children and adolescents^30–33^. This is in line with results of the broad, multi-center Polish study (2005–2013) showing the excessive body weight in 8.4% of children newly diagnosed with IBD. According to that report, however, excessive body weight was three times more common in newly diagnosed UC than in CD^11^.

The importance of obesity for the IBD course in children and adolescents has not yet been fully understood, and the literature data are mixed^12,34,35^. Excessive adiposity has been previously related to proinflammatory state and increased gut permeability as well^9^. The study by von Graffenried et al. (2022) revealed obese children with IBD reported arthritis and perianal abscesses more often than under- and normal-weighted patients, but there was no difference in the activity and severity of IBD regarding the body weight status. IBD-related surgeries were mostly performed in underweight and overweight patients, though^35^. Therefore, relationship between the disease severity and BMI may be U-shaped. Cohen and coworkers pointed out that IBD patients with both low and high BMI percentile position were at higher risk of extraintestinal manifestation^34^, which has been associated with more severe IBD course^36^. In our study univariate analysis showed BMI and subcutaneous fat content were greater in CD patients with EIMs and PA disease. However, when controlling the duration of the disease, the presence of EIMs and PA disease tended to decrease the indicators of nutritional status.

Indeed, among our CD patients, the duration of the disease occurred primarily related to the nutritional status indicators, when in fact such a relationship has been poorly discussed in the literature so far. Single studies in children with IBD implied no link between the disease duration before diagnosis and anthropometry^8,11,27^, or showed longer disease duration in patients with both lowest and highest BMI z-score quartiles at diagnosis^10^. It must be emphasized, though, all the mentioned works have analyzed multiple IBD entities combined, with no specific reference to CD cases.

We have also found positive correlation between the age of newly diagnosed CD children and the abdominal SFT, and (slightly) the sum of SFTs. Nevertheless, disease duration has remained much stronger correlate with the sum of SFTs, as well as with many other nutritional indicators. Data on the association between body weight and the age at IBD diagnosis are incoherent. Study among children newly diagnosed with CD showed that obese ones were younger at diagnosis than other patients^12^, while studies comprising both IBD entities reported positive^9^, negative^27^ or no correlation between BMI z-scores and the age at diagnosis^29^.

In summary, among our CD (but not UC) patients, we found a relation between longer disease duration, better nutritional status, and the presence of EIMs and PA disease. Therefore, we speculate that the lack of visible body weight and body fat deficits combined with symptoms outside the gastrointestinal tract might delay the diagnosis of CD, especially if there are no or neglectable gastrointestinal symptoms. Children with no red flags among their symptoms can be often misdiagnosed as irritable bowel syndrome^37^. Three studies based on Spanish, Canadian and German-Austrian registries revealed greater (about twofold) diagnostic delay in pediatric CD compared to UC. In these studies, the diagnostic delay, among many factors, was related to the isolated small bowel disease and to the presence of perianal abscesses, which remains consistent with our results. Logically, earlier diagnosis was linked to worse indices of clinical disease activity, higher values of inflammatory markers, bloody diarrhea, vomiting, fever, body weight loss in IBD, and with nocturnal abdominal pain in UC^5,38,39^. The presence of solely, or mainly, EIMs as aphthous stomatitis, arthritis, skin lesions or pancreatic conditions was more often observed in CD than in UC, and significantly delayed the diagnosis^1,36^.

In our patients diagnosed with UC, only the presence of anemia was related to lower body fatness and to modestly lower BMI. Importantly, body weight loss was four times more common in anemic children with UC than in those without anemia. Specific symptoms as diarrhea, blood, and mucus in stool^1^ might be related to red blood cells loss and micro- and macronutrients deficiencies, leading further to the body weight loss and deficits, and anemia as well. Such an overt disease manifestation shortens the time from the first symptoms to diagnosis in UC^1,5^, which might explain only slight decrease in the anthropometric indices of nutritional status compared to the standard.

The study has some limitations. Clinical data were obtained retrospectively, thus certain records became unavailable, leading to minor gaps. For this reason, we also could not include in the analysis other important variables as the level of fecal calprotectin and the phenotype of CD. Restrictive selection of patients considerably reduced the sample size, but in turn let us obtain the net effect of the disease on somatic indices, eliminating the potential influence of specific treatment or conditions disrupting growth and nutritional status. Of note, anthropometric methods we applied went way beyond those commonly used in similar works (such as body height, body weight and BMI), allowing us to assess body composition regardless of the hydration status, which is essential in patients with acute diarrhea. All measurements and indicators were transformed into z-scores solely based on the national standards.

Nutritional status of newly diagnosed patients with IBD is differently related to disease course regarding disease entity. In pediatric CD better BMI and greater subcutaneous fat content, accompanied with extraintestinal symptoms and/or PA disease, may delay the diagnosis due to the lack of red flags. In pediatric patients with UC only anemia, due to intestinal losses, is related to low BMI and body weight loss before diagnosis. In the era of growing incidence of overweight and obesity in pediatric population, physicians should be more aware of the risk of gastrointestinal diseases causing malnutrition in children with normal and excessive body weight.

### Supplementary Information

Supplementary Information 2.

## Data Availability

The data underlying this article will be shared on a reasonable request to the corresponding author.

## Abbreviations

BMI: Body mass index
CD: Crohn’s disease
EIMs: Extraintestinal manifestations
GI: Gastrointestinal
hsCRP: High-sensitivity C-reactive protein
IBD: Inflammatory bowel diseases
mo: Months
MUAC: Mid-upper arm circumference
MUAMC: Mid-upper arm muscle circumference
PA: Perianal
PCDAI: Pediatric Crohn’s Disease Activity Index
PUCAI: Pediatric Ulcerative Colitis Activity Index
SD: Standard deviation
SFT: Skinfold thickness
SI: Small intestine
UC: Ulcerative colitis
y: Years
