# Supplementary material for: Nutritional status of pediatric patients with inflammatory bowel diseases is related to disease duration and clinical picture at diagnosis
Source: Sci Rep. 2023 Dec 2;13:21300. doi: 10.1038/s41598-023-48504-8 (PMC10693555; doi:10.1038/s41598-023-48504-8)
Supplement: Supplementary file 1 — Supplementary Information 2. [file 41598_2023_48504_MOESM1_ESM.docx]

Supplemetary Information

**Nutritional status of pediatric patients with inflammatory bowel diseases is related to disease duration and clinical picture at diagnosis, K. Pawłowska-Seredyńska et al.**

**Table 1** Pearson’s correlation between studied qualitative features in patients with Crohn’s disease.

|  | Age at examination (y) | Age of the first symptoms (y) | Disease duration (mo) | hsCRP (mg/dL) | Albumin (mg/dL) | Total protein (mg/dL) | Total cholesterol (mg/dL) | HGB (mg/dL) | PCDAI score* |
| --- | --- | --- | --- | --- | --- | --- | --- | --- | --- |
| BH (z-score) | ns | ns | ns | ns | ns | ns | ns | ns | ns |
| BW (z-score) | ns | ns | r = 0.38  p = 0.017  95% CI: 0.07-0.62 | ns | ns | ns | ns | ns | r = -0.45  p = 0.006  95% CI: -0.68 – -0.14 |
| BMI (z-score) | ns | r = -0.37  p = 0.022  95% CI: | r = 0.42  p = 0.007  95% CI: 0.12-0.65 | ns | ns | ns | ns | ns | r = -0.46  p = 0.005  95% CI: -0.68 – -0.15 |
| Tricipital SFT (z-score) | ns | ns | r = 0.43  p = 0.006  95% CI: 0.13-0.66 | ns | ns | ns | ns | ns | r = -0.46  p = 0.005  95% CI: -0.68 – -0.15 |
| Subscapular SFT (z-score) | ns | ns | r = 0.57  p < 0.001  95% CI: 0.31-0.75 | ns | ns | ns | ns | r = 0.35  p = 0.031  95% CI: 0.03-0.60 | r = -0.41  p = 0.012  95% CI: -0.65 – -0.10 |
| Abdominal SFT (z-score) | r = 0.40  p = 0.009  95% CI: 0.10-0.63 | ns | ns | ns | r = 0.33  p = 0.036 95% CI: 0.02-0.58 | ns | ns | 0.34  P=0.033  95% CI: 0.03-0.59 | r = -0.41  p = 0.013  95% CI: -0.65 – -0.10 |
| Sum of SFTs (z-score) | r = 0.32  p = 0.042  95% CI: 0.01-0.57 | ns | r = 0.42  p = 0.007  95% CI: 0.12-0.65 | ns | r = 0.31  p = 0.049  95% CI: 0.00-0.57 | ns | ns | 0.35  P=0.031 95% CI: 0.35-0.60 | r = -0.44  p = 0.007  95% CI: -0.67 – -0.14 |
| MUAC (z-score) | ns | ns | ns | ns | ns | ns | ns | ns | r = -0.46  p = 0.005  95% CI: -0.68 – -0.15 |
| MUAMC (z-score) | ns | ns | ns | ns | ns | ns | ns | ns | ns |
| hsCRP (mg/dL) | ns | ns | ns | - |  |  |  |  |  |
| Albumin (mg/dL) | ns | ns | ns | r = -0.48  p = 0.002  95% CI: -0.69-(-0.20) | - |  |  |  |  |
| Total protein (mg/dL) | ns | ns | ns | ns | r = 0.54  p < 0.001 95% CI: 0.27-0.73 | - |  |  |  |
| Total cholesterol (mg/dL) | ns | ns | r = -0.41  p = 0.020  95% CI: -0.67-(-0.07) | ns | ns | ns | - |  |  |
| HGB (mg/dL) | ns | ns | ns | r = -0.37  p = 0.019 95% CI: -0.62-(-0.07) | r = 0.45  p = 0.004  95% CI: 0.16-0.67 | ns | ns | - |  |
| PCDAI score* | ns | ns | ns | r = 0.47  p = 0.005  95% CI: 0.15 – 0.69 | r = -0.61  p <0.001  95% CI: -0.35 – (-0.79) | na | na | ns | - |

BMI – body mass index, HGB – hemoglobin, hs CRP – high sensitivity C-reactive protein, mo – months, MUAC -mid-upper arm circumference, MUAMC – mid-upper arm muscle circumference, ns – not significant, PCDAI – Pediatric Crohn’s Activity Index, SFT – skinfold thickness, y – years.

*PCDAI with modification by Ryżko and Woynarowski (see Appendix 1, page … of Supplementary Information) includes Cole’s Index, therefore the significant correlation with nutritional status may be due to autocorrelation between studied variables.

**Table II** Mean values ± SD of age, age of the first symptoms, disease duration and sex- and age-adjusted anthropometric indices (z-scores) of: children with Crohn’s disease according to disease location (A), children with Crohn’s disease according to the type of symptoms (B), and children with ulcerative colitis regarding anemia (C) and statistical significances of differences in anthropometry between groups according to unpaired t-test or Mann-Whithey’ test.

| **Table IIa** | SI lesions (n=21) | No SI lesions (n=17) | p-value |
| --- | --- | --- | --- |
| Age at examination (y) | 13.34 ± 2.67 | 13.00 ± 3.21 | ns (t) |
| Age of the first symptoms (y) | 9.43 ± 2.67 | 11.42 ± 3.89 | ns (t) |
| Disease duration (mo)  Me and IQR | 47.31 ± 52.54  26.81, 12.15 – 51,70 | 18.87 ± 26.92  8.12, 4.50 – 12.76 | 0.017 (t)* |
| Body height (z-score) | -0.04 ± 1.31 | -0.50 ± 1.17 | ns (t) |
| Body weight (z-score) | -0.37 ± 1.17 | -1.12 ± 0.98 | 0.044 (t) |
| BMI (z-score) | -0.46 ± 1.02 | -1.15 ± 1.03 | 0.047 (t) |
| Triceps SFT (z-score) | 0.38 ± 1.15 | -0.33 ± 1.05 | 0.058 (t) |
| Subscapular SFT (z-score) | 0.28 ± 1.37 | -0.42 ± 0.88 | ns (Z) |
| Abdominal SFT (z-score) | 0.60 ± 1.51 | 0.45 ± 1.86 | ns (Z) |
| Sum of 3 SFTs (z-score) | 0.49 ± 1.40 | -0.07 ± 1.35 | ns (Z) |
| MUAC (z-score) | 0.01 ± 1.17 | -0.73 ± 1.07 | 0.055 (t) |
| MUAMC (z-score) | -0.23 ± 1.08 | -0.78 ± 1.16 | ns (t) |

BMI – body mass index, IQR – interquartile range, Me – median, mo – months, MUAC -mid-upper arm circumference, MUAMC – mid-upper arm muscle circumference, ns – not significant, SFT – skinfold thickness, SI – small intestinal, y – years.

(t) p-value according to unpaired t-test

(Z) p-value according to Mann-Whitney’ test

* logarithm data

| **Table IIb** | EIMs and PA disease (n=23) | GI symptoms only (n=17) | p-value |
| --- | --- | --- | --- |
| Age at examination (y) | 13.45 ± 2.87 | 12.98 ± 2.93 | ns (t) |
| Age of the first symptoms (y) | 9.85 ± 4.41 | 11.38 ± 4.04 | ns (t) |
| Disease duration (mo)  Me and IQR | 43.19 ± 52.35  24.03, 6.48 – 55.36 | 19.34 ± 20.66  11.88, 4.80 – 23.90 | ns (t)* |
| Body height (z-score) | -0.06 ± 1.36 | -0.57 ± 1.02 | ns (t) |
| Body weight (z-score) | -0.40 ± 1.20 | -1.19 ± 0.84 | 0.024 (t) |
| BMI (z-score) | -0.47 ± 1.07 | -1.29 ± 0.87 | 0.014 (t) |
| Triceps SFT (z-score) | 0.38 ± 1.23 | -0.43 ± 0.78 | 0.022 (t) |
| Subscapular SFT (z-score) | 0.25 ± 1.35 | -0.53 ± 0.80 | 0.032 (Z) |
| Abdominal SFT (z-score) | 0.85 ± 1.64 | 0.05 ± 1.52 | 0.066 (Z) |
| Sum of 3 SFTs (z-score) | 0.57 ± 1.41 | -0.31 ± 1.15 | 0.042 (t) |
| MUAC (z-score) | -0.02 ± 1.21 | -0.85 ± 0.92 | 0.028 (t) |
| MUAMC (z-score) | -0.25 ± 1.13 | -1.06 ± 1.27 | 0.042 (t) |

BMI – body mass index, EIMs – extraintestinal manifestations, GI – gastrointestinal, IQR – interquartile range, Me – median, mo – months, MUAC -mid-upper arm circumference, MUAMC – mid-upper arm muscle circumference, ns – not significant, PA – perianal, SFT – skinfold thickness, y – years.

(t) p-value according to unpaired t-test

(Z) p-value according to Mann-Whitney’ test

* logarithm data

| **Table IIc** | UC with anemia (n = 19) | UC with no anemia (n = 10) | p-value |
| --- | --- | --- | --- |
| Age at examination (y) | 13.35 ± 3.27 | 11.59 ± 3.05 | ns (t) |
| Age of the first symptoms (y) | 12.60 ± 3.40 | 10.75 ± 3.37 | ns (t) |
| Disease duration (mo)  Me and IQR | 8.99 ± 7.63  6.94, 2.24 – 12.82 | 10.06 ± 11.51  5.65, 3.32 – 12.33 | ns (t)* |
| Body height (z-score) | -0.11 ± 1.26 | 0.23 ± 0.95 | ns (t) |
| Body weight (z-score) | -0.68 ± 1.19 | 0.06 ± 1.24 | ns (t) |
| BMI (z-score) | -0.76 ± 0.90 | -0.14 ± 1.07 | ns (Z) |
| Triceps SFT (z-score) | -0.18 ± 0.81 | 0.38 ± 0.90 | ns (t) |
| Subscapular SFT (z-score) | -0.44 ± 0.66 | 0.43 ± 1.32 | 0.039 (Z) |
| Abdominal SFT (z-score) | 0.01 ± 0.75 | 0.98 ± 1.81 | ns (Z) |
| Sum of 3 SFTs (z-score) | -0.21 ± 0.76 | 0.69 ± 1.43 | 0.041 (Z) |
| MUAC (z-score) | -0.29 ± 1.17 | 0.07 ± 1.61 | ns (Z) |
| MUAMC (z-score) | -0.28 ± 1.27 | -0.18 ± 1.73 | ns (t) |

BMI – body mass index, IQR – interquartile range, Me – median, mo – months, MUAC -mid-upper arm circumference, MUAMC – mid-upper arm muscle circumference, ns – not significant, SFT – skinfold thickness, y – years.

(t) p-value according to unpaired t-test

(Z) p-value according to Mann-Whitney’ test

* logarithm data

**Table 3** Pearson’s correlation between studied qualitative features in patients with ulcerative colitis (n=29).

|  | Age at examination (y) | Age of the first symptoms (y) | Disease duration (mo) | hsCRP (mg/dL) | Albumin (mg/dL) | Total protein (mg/dL) | Total cholesterol (mg/dL) | HGB (mg/dL) | PUCAI score |
| --- | --- | --- | --- | --- | --- | --- | --- | --- | --- |
| Body height (z-score) | ns | ns | ns | ns | ns | ns | ns | ns | ns |
| Body weight (z-score) | ns | ns | ns | ns | ns | ns | ns | ns | ns |
| BMI (z-score) | ns | ns | ns | ns | ns | ns | ns | ns | ns |
| Tricipital SFT (z-score) | ns | ns | ns | ns | ns | ns | ns | ns | ns |
| Subscapular SFT (z-score) | ns | ns | ns | ns | ns | ns | ns | ns | ns |
| Abdominal SFT (z-score) | ns | ns | ns | ns | ns | ns | ns | ns | ns |
| Sum of SFTs (z-score) | ns | ns | ns | ns | ns | ns | ns | ns | ns |
| MUAC (z-score) | ns | ns | ns | ns | ns | ns | ns | ns | ns |
| MUAMC (z-score) | ns | ns | ns | ns | ns | ns | ns | ns | ns |
| hsCRP (mg/dL) | ns | ns | ns | - |  |  |  |  |  |
| Albumin (mg/dL) | ns | ns | ns | r = ­-0.44  p = 0.016  95% CI: -0.70-(-0.09) | - |  |  |  |  |
| Total protein (mg/dL) | ns | ns | ns | ns | r=0.58  p=0.001  95% CI: 0.26-0.78 | - |  |  |  |
| Total cholesterol (mg/dL) | ns | ns | ns | ns | ns | ns |  |  |  |
| HGB (mg/dL) | ns | ns | ns | r = -0.65  p < 0.001  95% CI: -0.82-(-0.37) | r = 0.37  p = 0.050  95% CI: 0.00-0.65 | ns | ns | - |  |
| PUCAI score | ns | ns | ns | ns | r = -0.74  p < 0.001  CI: -0.88 – (-0.47) | ns | ns | ns | - |

BMI – body mass index, HGB – hemoglobin, hs CRP – high sensitivity C-reactive protein, mo – months, MUAC -mid-upper arm circumference, MUAMC – mid-upper arm muscle circumference, ns – not significant, PUCAI – Pediatric Ulcerative Colitis Activity Index, SFT – skinfold thickness, y - years.

**Appendix 1. The questionnaire of Pediatric Crohn’s Diseasese Activity Index (PCDAI) by Hyams modified by Ryżko and Woynarowski.**

| **Symptoms** | **Scores** |
| --- | --- |
| **Abdominal pain:**  None  Mild  Severe | **0**  **5**  **10** |
| **Stools per day:**  <2 with no blood  2-5 loose sools with small blood  >5 loose stools or gross blood | **0**  **5**  **10** |
| **General well-being:**  Well (no limitation of activities)  Below par (occasional difficulty of maintaining age-appropriate activities)  Very poor (frequent limitation of activity) | **0**  **5**  **10** |
| **Hemoglobin level (g/dL):**  >12  10-12  <10 | **0**  **5**  **10** |
| **ESR (mm/h):**  <20  20-50  >50 | **0**  **5**  **10** |
| **Albumin level (g/dL):**  >3.5  3.1-3.5  <3.1 | **0**  **5**  **10** |
| **Cole’s Index:**  >85  80-85  <80 | **0**  **5**  **10** |
| **Abdomen:**  No tenderness, no mass  Tenderness or mass without tenderness  Definite tenderness and mass | **0**  **5**  **10** |
| **Peri-rectal disease:**  None  Indolent, no tenderness  Active fistula, tenderness, or abscess | **0**  **5**  **10** |
| **Extraintestinal manifestations:**  None  1  ≥2 | **0**  **5**  **10** |

**Sources:**

Ryżko, J. & Woynarowski, M. Ocena przebiegu nieswoistych zapaleń jelit u dzieci według systemu punktowego. *Pediatria Polska* **70**, 569–573 (1995).

Ryżko, J. & Woynarowski, M. Zastosowanie skali punktowej w ocenie aktywności choroby Leśniowskiego-Crohna i wrzodziejącego zapalenia jelita grubego u dzieci. *Pediatria Polska* **70**, 585–589.
